# Differential modulation of short-term plasticity at hippocampal mossy fiber and Schaffer collateral synapses by mitochondrial Ca^2+^

**DOI:** 10.1371/journal.pone.0240610

**Published:** 2020-10-13

**Authors:** Sang Hun Lee, David Lutz, Dagmar Drexler, Michael Frotscher, Jie Shen

**Affiliations:** 1 Department of Neurology, Brigham & Women’s Hospital, Harvard Medical School, Boston, Massachusetts, United States of America; 2 Institute for Structural Neurobiology, Center for Molecular Neurobiology Hamburg (ZMNH), University Medical Center Hamburg-Eppendorf, Hamburg, Germany; 3 Program in Neuroscience, Harvard Medical School, Boston, Massachusetts, United States of America; Nathan S Kline Institute, UNITED STATES

## Abstract

Presynaptic mitochondrial Ca^2+^ plays a critical role in the regulation of synaptic transmission and plasticity. The presynaptic bouton of the hippocampal mossy fiber (MF) is much larger in size than that of the Schaffer collateral (SC) synapse. Here we compare the structural and physiological characteristics of MF and SC presynaptic boutons to reveal functional and mechanistic differences between these two synapses. Our quantitative ultrastructural analysis using electron microscopy show many more mitochondria in MF presynaptic bouton cross-section profiles compared to SC boutons. Consistent with these results, post-tetanic potentiation (PTP), a form of presynaptic short-term plasticity dependent on mitochondrial Ca^2+^, is reduced by inhibition of mitochondrial Ca^2+^ release at MF synapses but not at SC synapses. However, blockade of mitochondrial Ca^2+^ release results in reduction of PTP at SC synapses by disynaptic MF stimulation. Furthermore, inhibition of mitochondrial Ca^2+^ release selectively decreases frequency facilitation evoked by short trains of presynaptic stimulation at MF synapses, while having no effect at SC synapses. Moreover, depletion of ER Ca^2+^ stores leads to reduction of PTP at MF synapses, but PTP is unaffected by ER Ca^2+^ depletion at SC synapses. These findings show that MF and SC synapses differ in presynaptic mitochondrial content as well as mitochondrial Ca^2+^ dependent synaptic plasticity, highlighting differential regulatory mechanisms of presynaptic plasticity at MF and SC synapses.

## Introduction

The hippocampus is divided into three main fields, the dentate gyrus (DG) and areas CA3 and CA1, and each field displays distinctive anatomical, molecular, and biophysical properties [1, 2]. The tri-synaptic circuit mediates the signal flow through the hippocampus and consists of three excitatory synaptic pathways: perforant path → DG, mossy fiber (MF) → CA3, and Schaffer collateral (SC) → CA1 [3]. The mechanism of synaptic transmission at the SC-CA1 synapse has been widely studied in relation to learning and memory [4–6], whereas the MF-CA3 projection has been implicated in cognitive function, including novelty detection, pattern completion and partially pattern separation [7, 8]. In addition, MF synapses between granule cells of the dentate gyrus and pyramidal neurons of the CA3 region are known to exhibit unique ultrastructural characteristics compared with other hippocampal synapses, and their presynaptic terminals are called ‘giants’ boutons [9]. In contrast to SC synapses, MF synapses display low basal neurotransmitter release probability, resulting in pronounced paired-pulse facilitation (PPF), frequency facilitation, and post-tetanic potentiation (PTP), which are mediated by intra-bouton accumulation of residual calcium during repetitive presynaptic stimulation [10–14].

Proper axonal transport and synaptic distribution of mitochondria and/or endoplasmic reticuli (ER) has been shown to play a crucial role in the maintenance of synaptic homeostasis during neuronal activity. Indeed, electron microscopy studies have provided evidence for the presence of mitochondria and ER at MF terminals [15]. Presynaptic mitochondria and ER contribute to the regulation of synaptic transmission and plasticity by sequestering Ca^2+^, thereby accelerating functional recovery during periods of moderate-to-high presynaptic activity [16]. Importantly, disruptions of mitochondrial or ER Ca^2+^ homeostasis at presynaptic axon terminals results in aberrant synaptic transmission [17–20]. Many neurodegenerative diseases, including Alzheimer’s, Parkinson’s, Huntington’s, and amyotrophic lateral sclerosis, involve defects in mitochondrial and/or ER function and transport [21–25].

In the present study, we investigate and compare the physiological characteristics of hippocampal MF and SC synapses. Using quantitative electron microscopy (EM) analysis, we found much greater numbers of mitochondria at MF terminals in contrast to very few mitochondria at SC boutons. Moreover, the magnitude of PTP, a form of short-term presynaptic plasticity mediated in part by mitochondrial Ca^2+^ release, is reduced upon inhibition of mitochondrial Na^+^/Ca^2+^ exchanger (NCX) at MF synapses but not at SC synapses. Interestingly, inhibition of mitochondrial Ca^2+^ release diminishes PTP at SC synapses by disynaptic MF stimulation. Similar to the effects on PTP, blockade of mitochondrial Ca^2+^ release decreases frequency facilitation at MF synapses selectively, whereas it has no effect at SC synapses. Furthermore, depletion of ER Ca^2+^ stores results in reduction of PTP at MF synapses, but PTP is unaffected by ER Ca^2+^ depletion at SC synapses. These findings show that MF and SC synapses differ in presynaptic mitochondrial content as well as mitochondrial Ca^2+^ dependent synaptic plasticity, highlighting differential regulatory mechanisms of presynaptic plasticity at MF and SC synapses.

## Materials and methods

### Mice

All experimental procedures were approved by the IACUC committees of Harvard Medical School and Brigham and Women's Hospital, and conform to the USDA Animal Welfare Act, PHS Policy on Humane Care and Use of Laboratory Animals, the "ILAR Guide for the Care and Use of Laboratory Animals" and other applicable laws and regulations. Male B6/129 mice at 2 months of age, were used throughout the study. All mice were housed in humidity- and temperature-controlled rooms maintained on a 12:12h light: dark cycle and were given standard rodent chow and water.

### Preparation of hippocampal slices

Mice were decapitated after being anesthetized with ketamine (100 mg/kg) + xylazine (10 mg/kg) + acepromazine (3 mg/kg), and the whole brains rapidly removed and placed in ice-cold (4°C) oxygenated (95% O_2_/5% CO_2_) high sucrose and magnesium solution containing (in mM) the following: 200 Sucrose, 25 NaHCO_3_, 10 Glucose, 3 KCl, 1.25 NaH_2_PO_4_, 1.2 Na-pyruvate and 0.4 Na-ascorbate, 7 MgCl_2_, and 0.5 CaCl_2_. Horizontal hippocampal slices (400 μm thick) were prepared using a vibratome (VT1200S, Leica, Germany), and transferred to an incubation chamber having oxygenated artificial cerebrospinal fluid (ACSF) containing (in mM) the following: 125 NaCl, 3 KCl, 1.25 NaH_2_PO_4_, 1 MgCl_2_, 2 CaCl_2_, 25 NaHCO_3_, 10 Glucose, 1.2 Na-pyruvate and 0.4 Na-ascorbate, adjusted to 310 ± 5 mOsm (pH 7.4). The slices were allowed to recover at 34°C for 30 min and then placed in a recording chamber constantly perfused with heated ACSF (30 ± 1°C) and gassed continuously with 95% O_2_ and 5% CO_2_. The flow rates of bathing solution and the volume of the recording chamber for slices were 2.2 ml/min and 1.2 ml, respectively. Hippocampal slices were visualized using an upright microscope equipped with differential interference contrast (DIC) optics (BX51WI, Olympus, Japan). The DIC optics was used for visualization of neurons in the course of whole-cell recordings.

### Electrophysiological analysis

For extracellular field recordings, stimulation pulses were delivered with a stimulus isolation unit (World Precision Instruments, A365) using a unipolar metal microelectrode. Stimulus electrodes were positioned ~600 μm from the recording electrode in *stratum radiatum* (Schaffer collaterals) or the hilus adjacent to the dentate granule cell layer (mossy fibers). Field excitatory postsynaptic potentials (fEPSPs) were recorded in current-clamp mode with ACSF-filled patch pipettes (1.5–2 MΩ). All fEPSPs were recorded with a stimulation strength that yielded 30% of the maximal response. To ensure that MF responses were not contaminated by associational/commissural inputs, the metabotropic glutamate receptor agonist (2S,1′R,2′R,3′R)-2-(2,3-dicarboxycyclopropyl) glycine (DCG-4; 2 μM) was applied at the end of experiments to block MF responses selectively. Data were included only if responses were reduced by more than 80%. All recordings were performed with the GABA_A_ receptor antagonist bicuculline methiodide (10 μM) and NMDA receptor antagonist APV (50 μM) added to the ACSF. Data were collected with a MultiClamp 700B amplifier (Molecular Devices) and digitized at 10 kHz using the A/D converter DIGIDATA 1322A (Molecular Devices). Data were acquired and analyzed using a custom program written with Igor Pro software (Version 6.3; Wave-Metrics) and Clampfit (Version 10.3; Molecular device).

PTP was induced by high frequency stimulation (HFS: 16 pulses at 100 Hz, delivered 4 times at 0.33 Hz) at MF-CA3 synapses or SC-CA1 synapses. The stimulation protocol on PTP induction was adjusted to induce the peak PTP amplitude at ranging from 200% to 300% of the baseline responses. After baseline responses were collected every 5 sec for 3 min, HFS was applied to induce PTP and the time-dependent changes in the fEPSP slope were recorded. PTP was recorded in the presence or absence of CGP37157 (20 μM) or tetraphenylphosphonium (TPP^+^; 2 μM), inhibitors of mitochondrial Na^+^/Ca^2+^ exchanger (NCX), and CGP37157 or TPP^+^ treatment began 15 min before HFS and lasted during the PTP recording. For inhibition of SERCA Ca^2+^ pumps on the ER, PTP was examined after treatment with 2 μM of thapsigargin (TG) for 40 min in the slices. The magnitude of PTP was quantified as the average of the first three post-tetanic fEPSP slopes normalized to the mean baseline slopes. Paired-pulse facilitation (PPF) was measured as the ratio of the second fEPSP slope relative to the first fEPSP slope, evoked by two identical presynaptic stimuli. Synaptic facilitation was measured as the percentage of the fEPSP slope versus the first fEPSP slope at a given stimulus train in individual slices. To assess the effect of CGP37157 on synaptic facilitations, we performed 15 min pre-treatment.

For whole-cell patch clamp experiments, recording pipettes (3–5 MΩ) were filled with a solution containing (in mM) the following: 120 K-gluconate, 10 KCl, 20 HEPES, 4 MgATP, 0.3 NaGTP, 10 phosphocreatine, and 0.1 EGTA with the pH adjusted to 7.30 with KOH (295–300 mOsm). Excitatory postsynaptic currents (EPSCs) at MF or SC synapses were recorded from CA3-pyramidal cells (CA3-PCs) or CA1-pyramidal cells (CA1-PCs) in voltage-clamp mode at a holding potential of -60 or -70 mV respectively. The series resistance (Rs) after establishing whole-cell configuration was between 15 and 20 MΩ. Synaptic responses were evoked by extracellular stimulation via a stimulator (Stimulus Isolator A365; WPI) connected to a patch electrode filled with ACSF solution, and placed in the hilus adjacent to the dentate granule cell layer (mossy fibers) or *stratum radiatum* (Schaffer collaterals). The stimulus intensity was adjusted such that the baseline EPSC amplitude was in the range between 100 pA and 200 pA. EPSC recordings with >20% series resistance change were excluded from data analysis. At the end of each experiment, we examined the effect of DCG-4 (2 μM) to confirm that we had studied MF synapses.

### Quantitative EM analysis

For quantitative EM analysis of mitochondria at SC and MF synapses, four B6/129 male mice at the age of 2 months were used. Animals were anaesthetized and then transcardially perfused with physiological saline followed by fixative solution containing 1% glutaraldehyde and 4% paraformaldehyde in 0.1 M phosphate buffer (pH 7.4). Fixed brains were isolated and stored in fixative solution at 4°C overnight, washed in phosphate buffer, and sectioned coronally on a vibratome (Leica VT 10005) at a thickness of 200 μm. Dorsal hippocampi were carefully excised and post-fixed in 1% OsO_4_ for 30 minutes. After rinsing in distilled water and dehydration in an ascending series of ethanol (block-staining with 0.5% uranyl acetate in 70% ethanol) followed by propylene oxide, the hippocampi were embedded in Epon (Fluka) and hardened at 65°C for two days. Thin sections of 70 nm from *stratum lucidum* of area CA3 and *stratum radiatum* of area CA1 were cut on an ultratome (Leica Ultracut) and mounted on formvar-coated 50-mesh copper grids. Sections were post-stained with lead citrate and subjected to electron microscopy (6,200× magnification, Philips CM100 electron microscope). To avoid multiple measurements of the same cross-sectional bouton profile in CA1 and CA3, randomized sections 5 μm apart from each other were analyzed and at least 10 micrographs per mouse in the cohorts of four animals per condition were used. The number of mitochondria per cross-sectional bouton profile and the cross-sectional bouton profile area were quantified.

### Statistical analysis

Statistical analysis was performed using two-tailed unpaired Student’s *t*-test for significance for the quantitative EM analysis. Paired Student’s *t*-test or two-way repeated-measures ANOVA with Bonferroni correction were used for all comparisons of the electrophysiological results. Unpaired Student’s *t*-test was used for comparisons of the mitochondrial or ER Ca^2+^ contribution to PTP induction in the MF versus SC synapses. All data are presented as mean ± SEM.

## Results

### More mitochondria at presynaptic MF terminals compared with SC terminals

**Fig 1:**
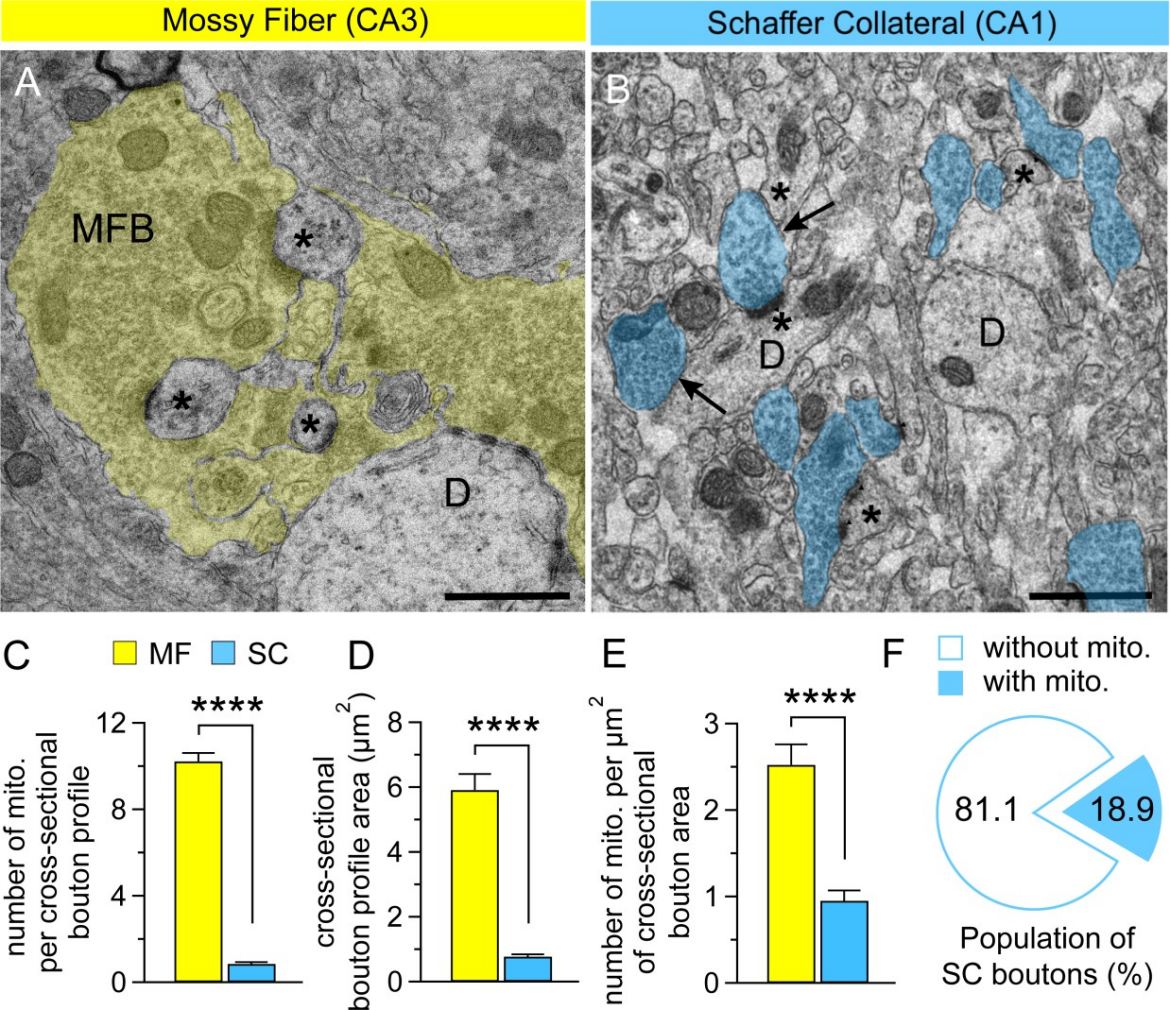
Comparison of mitochondrial number at MF and SC presynaptic bouton profiles. (**A**) A transmission electron micrograph of hippocampal *stratum lucidum* in area CA3 showing large cross-sectional mossy fiber bouton profiles (MFB: highlighted in yellow) establishing synaptic contacts with large complex spines of CA3 pyramidal neuron dendrites at multiple sites. Asterisk: spine, D: dendrite, Scale bar: 1 μm. (**B**) A transmission electron micrograph of hippocampal *stratum radiatum* in area CA1 showing cross-sectional dendrite profiles of CA1 pyramidal neurons and the neuropil in-between, which contains abundant cross-sectional presynaptic bouton and dendritic spine profiles. The cross-sectional areas of the bouton profiles are highlighted in blue. Arrows indicate cross-sectional bouton profiles with or without mitochondria. Asterisk: spine, D: dendrite, Scale bar: 1 μm. (**C**) Quantification of the number of mitochondria per cross-sectional bouton profile. Note that the cross-sectional MF bouton profiles contain many mitochondria, in contrast to the scarcity of mitochondria in the cross-sectional SC bouton profiles (MF: 10.22 ± 0.39, n = 45; SC: 0.84 ± 0.08, n = 45; MF *vs*. SC, *p* < 0.0001, unpaired *t*-test). (**D**) Quantification of the size of cross-sectional bouton profile area. The cross-sectional MF bouton profile areas are larger relative to the cross-sectional areas of SC bouton profiles (MF: 5.91 ± 0.49 μm^2^, n = 48; SC: 0.77 ± 0.07 μm^2^, n = 48; MF *vs*. SC, *p* < 0.0001, unpaired *t*-test). (**E**) Quantification of the number of mitochondria per μm^2^ of cross-sectional bouton area. The density of mitochondria is higher at MF boutons than SC boutons (MF: 2.52 ± 0.23 mito/μm^2^, n = 42; SC: 0.95 ± 0.12 mito/μm^2^, n = 44; MF *vs*. SC, *p* < 0.0001, unpaired *t*-test). (**F**) The fraction of cross-sectional CA1 bouton profiles containing or lacking mitochondria. The data are presented as mean ± SEM (**** *p* < 0.0001, Student’s *t*-test).

Presynaptic MF boutons are complex and form synaptic contacts at multiple sites with unique ultrastructural characteristics compared to other hippocampal synapses [9, 26, 27]. To determine whether the larger MF presynaptic boutons contain more mitochondria than the smaller SC presynaptic boutons, we performed quantitative EM analysis of both MF and SC presynaptic boutons in B6/129 wild-type mice at 2 months of age. Randomized samples from four mice were obtained and at least 10 representative micrographs of cross-sectional bouton profiles were analyzed. We found that in their cross-sectional profiles MF boutons often contain multiple mitochondria, whereas mitochondria are rare in the much smaller SC bouton cross-sections (Fig 1A and 1B); the cross-sectional terminal boutons of MF contain 10.22 (mean) ± 0.39 (SEM) mitochondria per cross-sectional profile (n = 45 analyzed cross-sectional bouton profiles), compared to the much lower number of mitochondria SC terminals per cross-sectional profile (0.84 ± 0.08, n = 45; MF *vs*. SC, *p* < 0.0001, unpaired *t*-test; Fig 1C and S1 Table). In addition, the cross-sectional MF bouton profile areas are larger (5.91 ± 0.49 μm^2^, n = 48) relative to the cross-sectional areas of SC bouton profiles (0.77 ± 0.07 μm^2^, n = 48; MF *vs*. SC, *p* < 0.0001, unpaired *t*-test; Fig 1D and S1 Table). Normalized on the cross-sectional bouton profile area, the density of mitochondria (2.52 ± 0.23 mito/μm^2^, n = 42) is higher at MF boutons than the density of mitochondria at SC boutons (0.95 ± 0.12 mito/μm^2^, n = 44; MF *vs*. SC, *p* < 0.0001, unpaired *t*-test; Fig 1E and S1 Table). Interestingly, most of the cross-sectional CA1 terminal profiles contain no mitochondria (profiles without mitochondria 81.1% versus 18.9% of mitochondria-containing profiles, n = 106; see Fig 1F). We also observed that the cross-sectional MF terminal profiles have formed multiple synaptic contacts with dendritic spines, while the SC terminals have mostly a single contact with a defined dendritic spine. These results indicate that relative to hippocampal SC boutons the larger MF boutons are much more versatile in ultrastructure and contain many more mitochondria.

### Presynaptic mitochondrial and ER Ca^2+^ contributes to PTP at MF synapses but not at SC synapses

**Fig 2:**
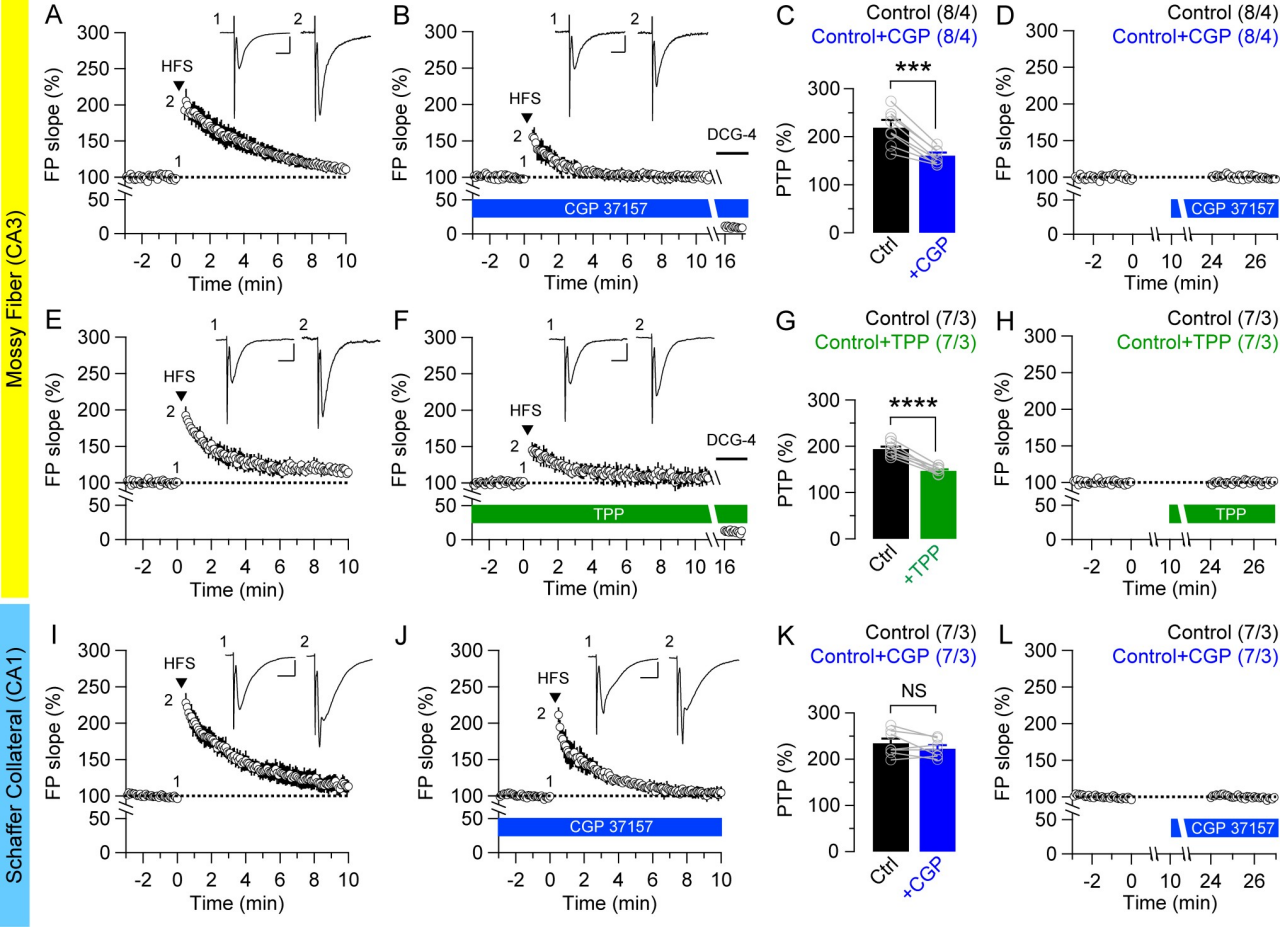
Differential effects of mitochondiral NCX inhibitors on PTP at MF and SC synapses. PTP was induced by high frequency stimulation (HFS: 16 pulses at 100 Hz, 4 times delivered at 0.33 Hz) in acute hippocampal slices of wild-type mice at 2 months of age. (**A** & **B**) Mean value of time-dependent changes in the fEPSP slope was recorded in the absence (A) or presence (B) of CGP37157 (20 μM), a mitochondrial NCX inhibitor, in the hippocampal MF pathway. The insets represent traces recorded before and immediately after HFS. DCG-4 (2 μM) was applied at the end of all experiments to confirm the recording of MF synapses. (**C**) The summary bar graph for the mean magnitude of PTP shows that PTP is reduced by CGP37157 treatment at MF synapses (Control: 219.1 ± 12.0%; +CGP: 157.1 ± 5.2%; *p* = 0.00013, paired *t*-test). (**D**) The effect of CGP37157 on basal transmission. Treatment of CGP37157 for 15 min has no effect on the fEPSP slope at MF synapses. (**E** & **F**) Mean value of time-dependent changes in the fEPSP slope was recorded in the absence (E) or presence (F) of TPP^+^ (2 μM), another specific inhibitor of mitochondrial NCX, in the hippocampal MF pathway. (**G**) The summary bar graph for the mean magnitude of PTP further shows that PTP induction is reduced at MF synapses (Control: 194.0 ± 5.2%; +TPP: 147.2 ± 2.7%; *p* < 0.0001, paired *t*-test). (**H**) The effect of TPP^+^ on basal transmission. Treatment of TPP^+^ for 15 min has no effect on the fEPSP slope at MF synapses. (**I** & **J**) Mean value of time-dependent changes in the fEPSP slope before and after the PTP induction in the hippocampal SC pathway. The insets represent traces recorded before and immediately after HFS. The effect of treatment with CGP37157 on PTP of fEPSPs at the hippocampal SC pathway shows that inhibition of mitochondrial Ca^2+^ release had no effect on PTP induction. (**K**) The summary bar graph for the mean magnitude of PTP shows that PTP induction is not different at SC synapses in the absence or the presence of CGP37157 treatment (Control: 234.7 ± 9.8%; +CGP: 222.7 ± 6.9%; *p* = 0.17, paired *t*-test). (**L**) The effect of CGP37157 on basal transmission. Treatment of CGP37157 for 15 min has no effect on the fEPSP slope at SC synapses. All data represent mean ± SEM (*** *p* < 0.001, **** *p* < 0.0001, NS: not significant; Student’s *t*-test). The value in parentheses indicates the number of hippocampal slices (left) and the number of mice (right) used in each experiment. Scale bar: 10 ms, 0.5 mV.

To investigate whether the observed differences in mitochondria content are translated into functional differences between these two hippocampal synapses, we performed field and whole-cell recording in acute hippocampal slices. Post-tetanic potentiation (PTP) is a form of short-term synaptic plasticity, lasting up to several minutes, and is longer-lasting than frequency facilitation, which is observed over several seconds. PTP is known to require mitochondrial Ca^2+^ for its induction and expression [10, 28–30]. First, we examined PTP at MF synapses in the presence of the NMDA receptor antagonist, D-APV (50 μM), and GABA_A_ receptor antagonist, bicuculline (10 μM), at hippocampal MF synapses. After recording the baseline fEPSPs for 3 min, PTP was induced by trains of high frequency stimulation (HFS: 16 pulses at 100 Hz, delivered 4 times at 0.33 Hz), and time-dependent changes in the fEPSP slope were measured. 15 min application of CGP37157 (20 μM), which inhibits mitochondrial Ca^2+^ release *via* the Na^+^/Ca^2+^ exchanger (NCX) [30, 31], in slices results in significant reduction of PTP in the MF pathway (Control: 219.1 ± 12.0%; +CGP: 157.1 ± 5.2%; *p* = 0.00013, paired *t*-test; Fig 2A–2C), suggesting that mitochondrial Ca^2+^ contributes to PTP induction at MF synapses. Using another specific inhibitor of mitochondrial NCX, tetraphenylphosphonium (TPP^+^; 2 μM, for 15 min) [32, 33], we found that TPP^+^ treatment also leads to a significant reduction of PTP in the MF pathway (Control: 194.0 ± 5.2%; +TPP: 147.2 ± 2.7%; *p* < 0.0001, paired *t*-test; Fig 2E–2G). These results confirm that inhibition of mitochondrial Ca^2+^ efflux reduces the magnitude of PTP, suggesting that mitochondrial Ca^2+^ at MF synapses contributes to PTP induction.

To determine whether the mitochondrial Ca^2+^-dependent PTP is specific to the hippocampal MF pathway, we conducted a similar series of experiments in the SC pathway. PTP at SC synapses in the presence of APV (50 μM) and bicuculline (10 μM) is similar to that at MF synapses (Fig 2I), however, unlike MF synapses, CGP37157 treatment had no effect on the magnitude of PTP at SC synapses (Control: 234.7 ± 9.8%; +CGP: 222.7 ± 6.9%; *p* = 0.17, paired *t*-test; Fig 2J and 2K). To compare the contribution of mitochondrial Ca^2+^ to PTP induction in MF versus SC synapses, we calculated the reduction percentage from the peak PTP levels in the absence or presence of blockers. After CGP37157 treatment, 27.4 ± 2.4% of PTP is decreased by mitochondrial Ca^2+^ inhibition in MF synapses while only 4.5 ± 2.8% of PTP is diminished at SC synapses (*p* = 0.00007, unpaired *t*-test; Table 1). These results indicate that the contribution of mitochondrial Ca^2+^ to PTP induction is negligible at SC synapses.

**Table 1:** Contributions of mitochondrial and ER Ca ^2+^ to PTP induction in the MF and SC synapses.

	Blockers	Reduction % of the peak PTP level after treatment of blocker (Monosynaptic effects)		*P* value of MF *vs*. SC (unpaired *t*-test)	Reduction % of the peak PTP level after treatment of blocker (Disynaptic effects: DG→CA1)
		MF synapse	SC synapse		
**Mito. Ca**^**2+**^	CGP37157	27.4 ± 2.4% (8/4)^a^	4.5 ± 2.8% (7/3)	0.00007	25.1 ± 2.2% (9/4)
	TPP^+^	23.9 ± 1.9% (7/3)	N/A^b^		N/A
**ER Ca**^**2+**^	Thapsigargin	30.4 ± 2.5% (6/3)	5.7 ± 1.8% (9/5)	0.000004	N/A

^a^The values in parentheses indicate the number of hippocampal slices (left) and the number of mice (right) used in each experiment.

^b^N/A: not applicable.

We also assessed the effects of CGP37157 or TPP^+^ on basal synaptic transmission at MF and SC synapses. Basal synaptic function was assayed by comparing the fEPSP slope of the second baseline (24–27 min) with the first baseline period (-3–0 min before adding CGP37157 or TPP^+^). We found that basal synaptic transmission is similar before and after the addition of CGP37157 or TPP^+^ at MF synapse (Fig 2D and 2H). Similarly, basal transmission does not differ before and after the CGP37157 treatment at SC synapses (Fig 2L).

**Fig 3:**
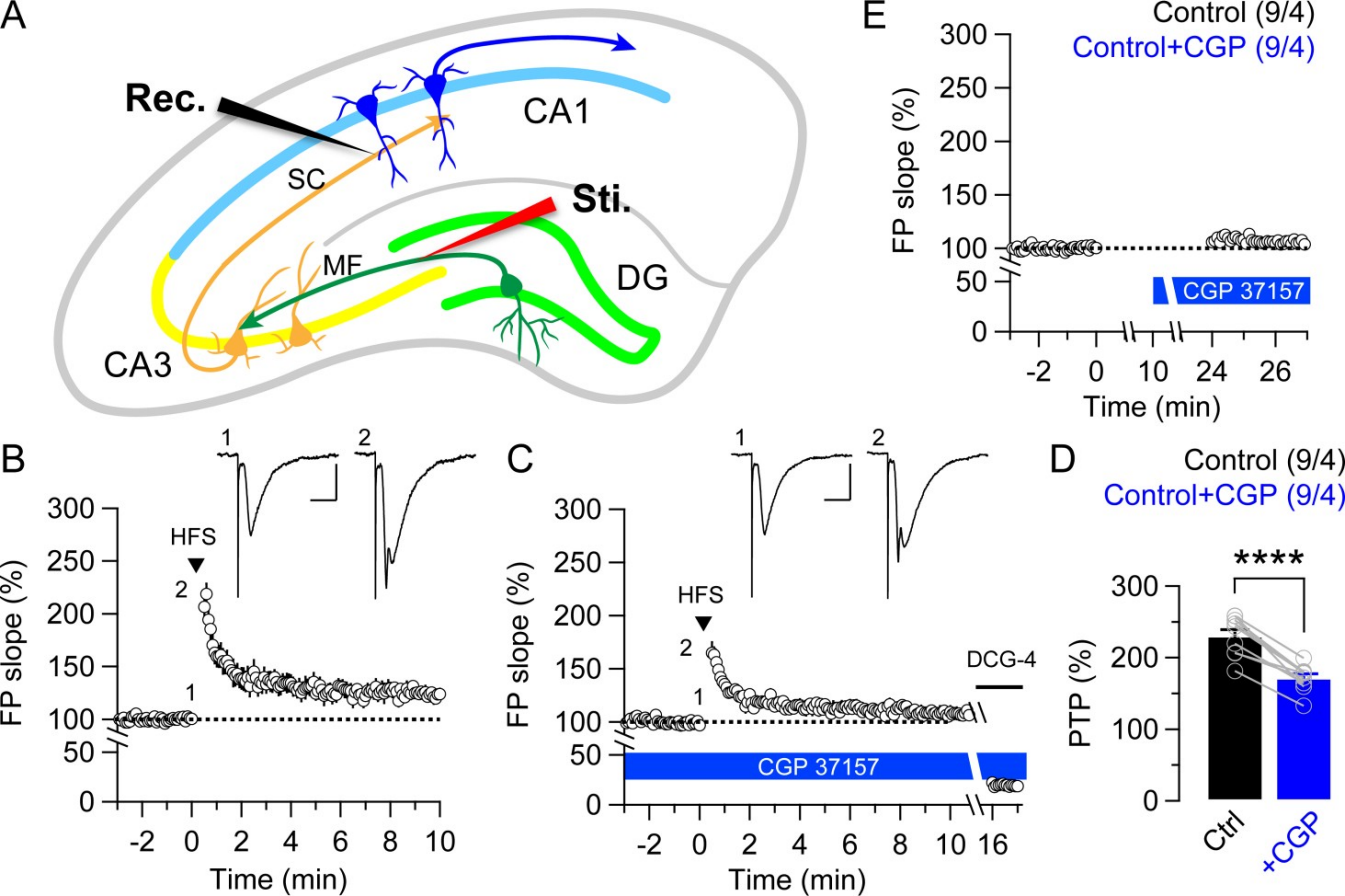
Inhibition of mitochondrial Ca^2+^ release reduces PTP at SC synapses by disynaptic MF stimulation. (**A**) A Schematic representation of a hippocampus showing the electrode location for stimulating (Sti.) MF synaptic input and recording fEPSPs (Rec.) in the *stratum radiatum*. (**B** & **C**) The magnitude of PTP induced by HFS is substantially less at SC synapses after 15 min treatment of CGP37157 (20 μM). The insets represent traces recorded before and immediately after HFS. Scale bar: 15 ms, 0.5 mV. DCG-4 (2 μM) was applied at the end of each experiment to confirm that MFs were stimulated. (**D**) Bar graphs show the mean magnitude of disynaptic PTP inductions before and after CGP37157 application (Control: 230.4 ± 8.7%; +CGP: 171.7 ± 5.8%; *p* < 0.0001, paired *t*-test). (**E**) The effects of CGP37157 treatment on basal transmission. The magnitude of basal transmission is similar in the absence or presence of CGP37157. All data are mean ± SEM (**** *p* < 0.0001; Student’s *t*-test). The value in parentheses indicates the number of hippocampal slices (left) and the number of mice (right) used in each experiment.

Our findings demonstrating the differences between MF and SC synapses on the mitochondrial Ca^2+^-dependent PTP are monosynaptic effects in the major hippocampal circuits. In contrast with the monosynaptic effects in which neurotransmissions are conducted via single synapses, disynaptic effects are transmitted via two synapses and intermediate neurons. To examine further the effects of blockade of mitochondrial Ca^2+^ release on the disynaptic pathway of hippocampal local-circuitry, we recorded fEPSPs at SC synapses by stimulating mossy fibers of dentate gyrus (DG) granule cells (Fig 3A). Interestingly, we found that the magnitude of PTP induced by trains of HFS is diminished after 15 min application of CGP37157 (20 μM) at SC synapses (Control: 230.4 ± 8.7%; +CGP: 171.7 ± 5.8%; *p* < 0.0001, paired *t*-test; Fig 3B–3D). Moreover, the contribution of mitochondrial Ca^2+^ to PTP induction is similar to that of monosynaptic MF responses (25.1 ± 2.2%; Table 1).

Basal synaptic response in disynaptic neurotransmission, however, is unchanged in the absence or presence of the mitochondrial NCX inhibitor (Fig 3E). These results reveal that the overall impact of the mitochondrial Ca^2+^ inhibition on PTP induction in this disynaptic pathway is to considerably reduce excitatory input to CA1 pyramidal neurons, thus demonstrating that the contribution of mitochondrial Ca^2+^ to synaptic transmissions at MF-CA3 synapses may dominate over the regulation of short-term plasticity at SC-CA1 synapses.

**Fig 4:**
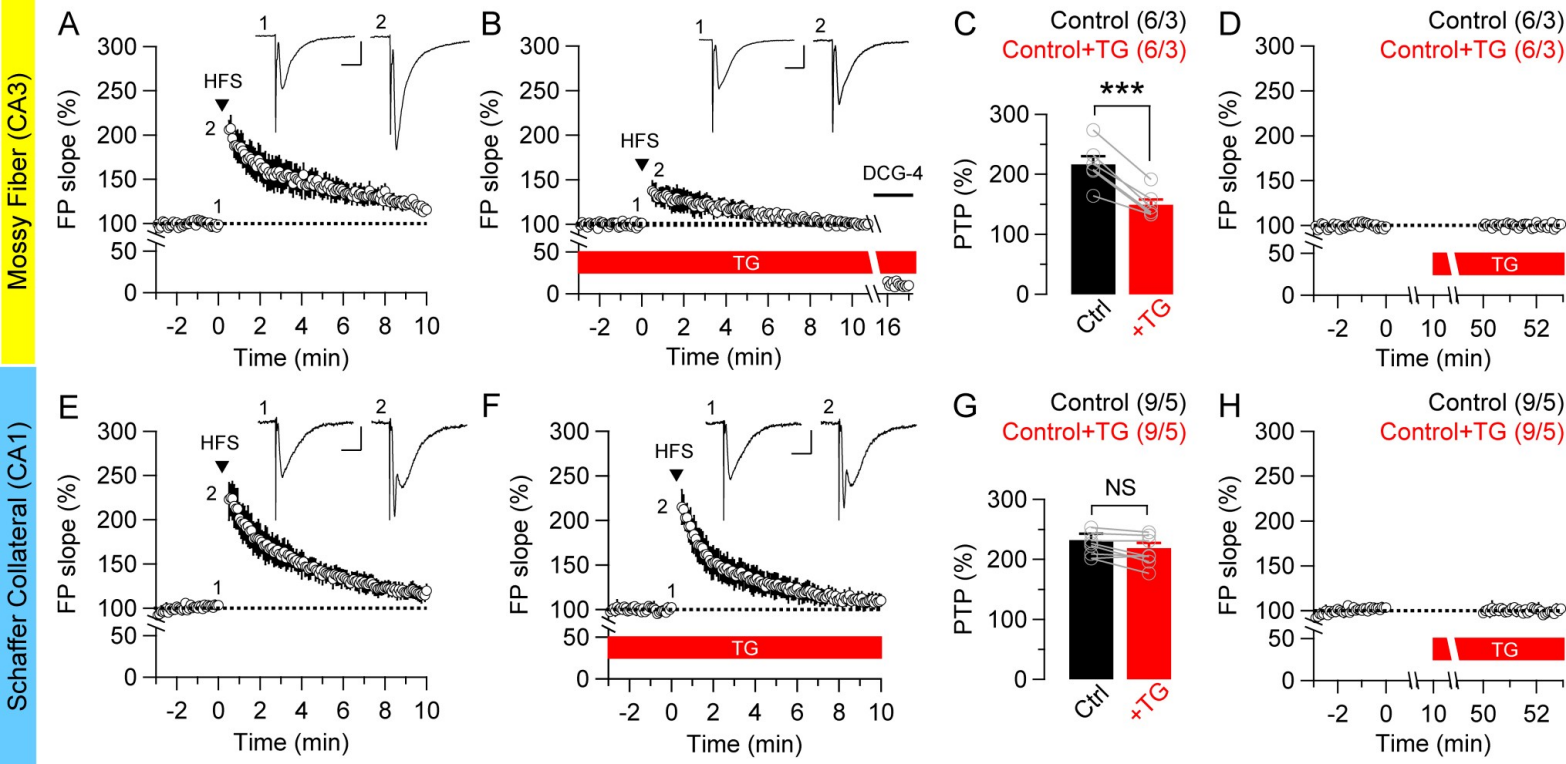
Depletion of ER Ca^2+^ differentially affects PTP at the hippocampal MF and SC synapses. (**A** & **B**) After applying thapsigargin (TG: 2 μM) for 40 min, which depletes ER Ca^2+^ by irreversibly blocks SERCA activity, the magnitude of PTP by HFS is substantially decreased at MF synapses. The insets represent traces recorded before and immediately after HFS. Scale bar: 10 ms, 0.5 mV. DCG-4 (2 μM) was applied at the end of all experiments to confirm the recording of MF synapses. (**C**) The summary bar graph for the mean magnitude of PTP induction in the absence or presence of TG at MF synapses (Control: 216.7 ± 13.4%; +TG: 149.8 ± 8.4%; *p* = 0.00045, paired *t*-test). (**D**) Effects of TG treatment on basal transmission. Treatment of TG for 40 min has no effect on the fEPSP slope at MF synapses. (**E** & **F**) Similarly, mean value of time-dependent changes in the fEPSP slope was recorded in the absence (E) or presence (F) of TG at hippocampal SC synapses. Note that TG treatment has no effect on PTP induction. (**G**) The summary bar graph for the mean magnitude of PTP induction in the presence of TG at SC synapses (Control: 232.5 ± 9.9%; +TG: 218.7 ± 8.8%; *p* = 0.34, paired *t*-test). (**H**) Effects of TG treatment on basal transmission. Treatment of TG for 40 min has no effect on the fEPSP slope at SC synapses. All data are mean ± SEM (*** *p* < 0.001, NS: not significant; Student’s *t*-test). The value in parentheses indicates the number of hippocampal slices (left) and the number of mice (right) used in each experiment.

We further examined whether blockade of ER Ca^2+^ release may differentially regulate synaptic plasticity at MF and SC synapses since physical interactions between ER and mitochondria are thought to facilitate their Ca^2+^ transfer [34–36]. We assessed the magnitude of PTP in the presence or absence of thapsigargin (TG), which irreversibly blocks the SERCA Ca^2+^ pumps and depletes Ca^2+^ in ER. After the treatment of slices with TG (2 μM) for 40 min, PTP is decreased at MF synapses (Control: 216.7 ± 13.4%; +TG: 149.8 ± 8.4%; *p* = 0.00045, paired *t*-test; Fig 4A–4C). Unlike MF synapses, TG treatment has no effect on PTP at SC synapses (Control: 232.5 ± 9.9%; +TG: 218.7 ± 8.8%; *p* = 0.34, paired *t*-test; Fig 4E–4G). The contribution of ER Ca^2+^ to PTP induction is 30.4 ± 2.5% at MF synapses and 5.7 ± 1.8% at SC synapses (*p* = 0.000004, unpaired *t*-test; Table 1).

We also examined the effects of TG on basal transmission at MF and SC synapses. The basal synaptic transmission was assayed by comparing the fEPSP slope of the second baseline (50–53 min) with the first baseline period (-3–0 min before adding TG). We confirmed that basal transmission is unaffected by the TG treatment at both MF and SC synapses (Fig 4D and 4H).

### Mitochondrial Ca^2+^ contribution to frequency facilitation at MF synapses but not at SC synapses

**Fig 5:**
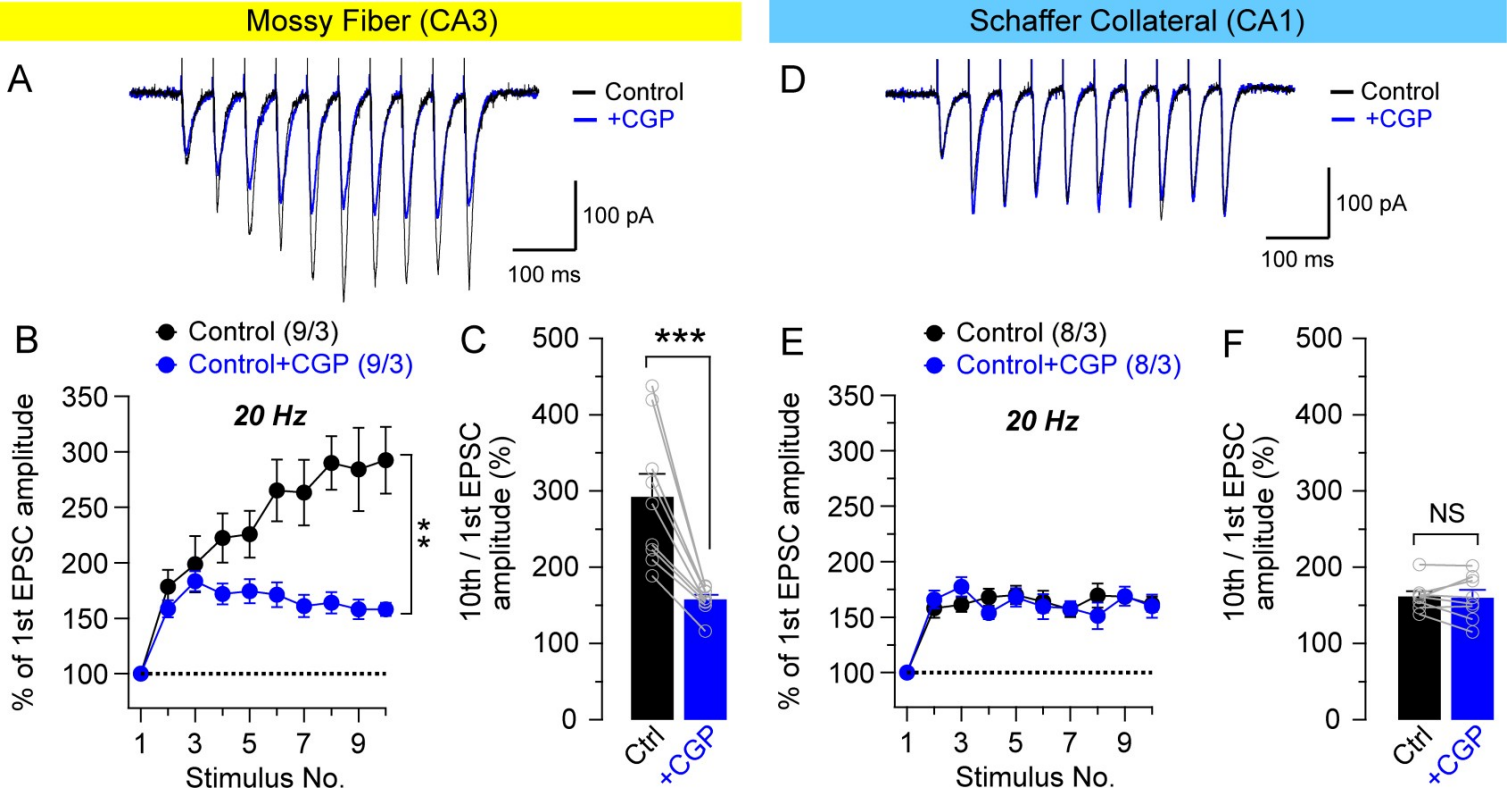
Inhibition of mitochondrial Ca^2+^ release selectively reduces synaptic facilitation at MF synapses. (**A**) Representative EPSCs recorded before and after treatment of CGP37157 (20 μM) on frequency facilitation elicited by 20 Hz stimulus train at MF synapses. Scale bar: 100 ms, 100 pA. (**B**) Inhibition of mitochondrial Ca^2+^ release by CGP37157 significantly reduces synaptic facilitation at MF synapses (F_1, 16_ = 10.23, *p* = 0.0056; two-way ANOVA). (**C**) Summary graph of the mean magnitude of the 10th stimulus train of frequency facilitation at MF synapses. The inhibition of mitochondrial Ca^2+^ release by CGP37157 significantly reduces the mean magnitude (157.9 ± 6.0%), relative to the control (292.4 ± 30.1%, *p* = 0.0008; paired *t*-test). (**D**) Representative EPSCs recorded before and after treatment of CGP37157 (20 μM) on frequency facilitation elicited by 20 Hz stimulus train at SC synapses. Scale bar: 100 ms, 100 pA. (**E**) Inhibition of mitochondrial Ca^2+^ release by CGP37157 has no effect on synaptic facilitation at SC synapses. (**F**) Summary graph of the mean magnitude of the 10th stimulus train of frequency facilitation at SC synapses. Inhibition of mitochondrial Ca^2+^ release by CGP37157 does not significantly reduce the mean magnitude (159.9 ± 10.4%), relative to the control (161.7 ± 6.8%, *p* = 0.776; paired *t*-test). All data represent mean ± SEM (** *p* < 0.01, *** *p* < 0.001, NS: not significant; two-way ANOVA for panels B and E, Student's *t*-test for panels C and F). The value in parentheses indicates the number of hippocampal neurons (left) and the number of mice (right) used in each experiment.

To determine whether presynaptic mitochondrial Ca^2+^ release also contributes to the regulation of frequency facilitation, induced by short trains of presynaptic stimulation (10 pulses), we examined frequency facilitation at 20 Hz in the absence or presence of CGP37157 at MF or SC synapses using whole-cell patch clamp recordings. Consistent with results obtained from field potential recording of PTP (Fig 2A–2C), we found that synaptic facilitation at 20 Hz is significantly reduced upon CGP37157 treatment at MF synapses (two-way ANOVA, F_1, 16_ = 10.23, *p* = 0.0056; Fig 5A and 5B). The mean magnitude of 10th stimulus train of the frequency facilitation displayed large reduction after the treatment with CGP37157 (157.9 ± 6.0%) relative to untreated controls (292.4 ± 30.1%; *p* = 0.0008, paired *t*-test; Fig 5C). These results suggest that mitochondrial Ca^2+^ contributes to synaptic facilitation at MF synapses.

At SC synapses, where the basal release probability is relatively high compared with MF, frequency facilitation (at 20 Hz) is less prominent (Fig 5D). To assess the contribution of mitochondrial Ca^2+^to PTP in these synaptic inputs, we assayed frequency facilitation in the presence of CGP37157. In contrast to MF synapses, we found that CGP37157 treatment had no effect on the frequency facilitation at SC synapses (two-way ANOVA, F_1, 14_ = 0.04, *p* = 0.84; Fig 5E). Consistently, inhibition of mitochondrial Ca^2+^ release by CGP37157 did not affect the mean magnitude of 10th stimulus train (159.9 ± 10.4%), relative to control (161.7 ± 6.8%; *p* = 0.776, paired *t*-test; Fig 5F). These results suggest that mitochondrial Ca^2+^ does not contribute significantly to synaptic frequency facilitation at hippocampal SC synapses.

## Discussion

A “giant” hippocampal MF bouton (3–10 μm in diameter) has been reported to contain 33–57 mitochondria [9], thus reflecting the high energy demand of its huge synaptic vesicle pool. We performed a direct comparison of the structural and functional properties of giant MF synapses from CA3 versus small SC synapses in area CA1 of the hippocampus. Based on our findings, the cross-sectional area of the MF bouton profiles appears to be almost eight times the size of the cross-sectional area of SC bouton profiles (Fig 1). In fact, ~81% of the presynaptic SC terminal profiles contain no mitochondria (Fig 1F). Consistent with these findings, contribution of mitochondrial Ca^2+^ to PTP induction and synaptic facilitation is negligible at SC synapses (Figs 2 and 5). However, the lack of mitochondria in 81% of SC boutons is based on analysis of single sections, thus we cannot exclude the possibility that some of the boutons did contain mitochondria at a different cross-sectional level. Because MF boutons are much larger than SC boutons, the chance of finding mitochondria in MFB cross sections seems to be higher. The ratio of mitochondria per bouton cross section area is 1.73 for MF synapses and 1.09 for SC synapses—still higher for MF synapses after correcting for the much larger surface area. These results are also consistent with earlier EM studies showing that over half of the axonal varicosities lack mitochondria in hippocampal CA1 *stratum radiatum* [37].

While the predominant functional role of mitochondria is energy metabolism, they are also involved in many other cellular processes relevant for neuronal functions, such as regulation of local Ca^2+^ homeostasis by acting as internal Ca^2+^ stores or regulating internal Ca^2+^ levels in the nerve terminal [38]. At presynaptic boutons, mitochondria may be involved in the mobilization of synaptic vesicles from the reserve pool [39]. Specifically, in MF boutons, mitochondria were seen to be associated with pools of synaptic vesicles (Fig 1A), suggesting their potential role in synaptic vesicle trafficking at multiple synaptic contact sites. Additionally, synaptic mitochondria have been shown to aid in critical physiological processes, including establishment of axonal resting membrane potential required for action potential propagation, assembly of actin cytoskeleton within presynaptic boutons [40], and myosin-driven mobilization of synaptic vesicles from the reserve pool to the readily releasable pool during sustained neuronal activity [18].

Furthermore, mitochondria’s ability to buffer Ca^2+^ within presynaptic terminals appears to be involved in certain types of short-term synaptic plasticity, such as PTP [16, 29, 41, 42]. PTP is known to be dependent on mitochondrial Ca^2+^ and is longer lasting than frequency facilitation due to the slower release of Ca^2+^ from mitochondria [28–30, 43]. Indeed, blockade of mitochondrial Ca^2+^ release by two different NCX inhibitors CGP37157 or TPP^+^ in hippocampal slices reduces the magnitude of PTP at MF synapses (Fig 2A–2H). These results are consistent with previous findings showing that PTP was diminished after treatment of mitochondrial Ca^2+^ inhibitors in the hippocampal MF synapse [30, 31, 33]. Thus, removal of mitochondria from axon terminals or functional blockade results in aberrant synaptic transmission [17, 18, 20]. Many neurodegenerative diseases, including Huntington’s disease, Parkinson’s disease, Alzheimer’s disease, and amyotrophic lateral sclerosis, involve defects in mitochondrial function and transport [22, 31, 33, 44, 45]. Interestingly, unlike MF synapses, blockade of mitochondrial Ca^2+^ release has no effect on PTP induction at SC synapses, indicating that mitochondrial Ca^2+^ contribution to PTP at SC synapses is negligible (Fig 2I–2L). Moreover, CGP37157 treatment impairs frequency facilitation at 20 Hz in MF synapses but has no effect on SC synapses, thus suggesting that mitochondrial Ca^2+^ also contributes to the synaptic facilitation at medium frequencies of the stimulus train at MF synapses but not at SC synapses (Fig 5). Indeed, a previous study reported that blockade of mitochondrial Ca^2+^ uptake by addition of Ru360 in the whole-cell patch pipette had no effect on Ca^2+^ decay rate and Ca^2+^ clearance in CA1 pyramidal neurons while Ca^2+^ dynamics was decreased in DG granule cells, indicating that mitochondrial contribution to Ca^2+^ homeostasis may be negligible in CA1 neurons [33]. Therefore, the differential effect of mitochondrial Ca^2+^ blockade on presynaptic short-term plasticity between hippocampal MF and SC synapses may be due to the difference of mitochondrial Ca^2+^ capacities based on their mitochondrial content.

ER Ca^2+^ stores play a major role in the regulation of synaptic facilitation at hippocampal synapses [46–49]. ER-mitochondria communication and the physical interaction of their membranes facilitates Ca^2+^ uptake from IP_3_ and ryanodine receptors on the ER by voltage-dependent anion channel and Ca^2+^ uniporter on mitochondria [34–36]. For example, when functional coupling between IP_3_ receptors and mitochondria is abolished, normal Ca^2+^ flow from the ER to mitochondria is reduced [50], whereas the transfer of Ca^2+^ to mitochondria is prolonged when IP_3_ receptors are stabilized [51]. Furthermore, the proximity between the ER and the mitochondrial outer membrane is crucial for the efficient transfer of Ca^2+^ [52, 53]. Such contacts, which represent mitochondria associated ER membranes, allow the bidirectional exchange of phospholipids and Ca^2+^ ions between both organelles [54, 55]. Consistently with these findings, depletion of ER Ca^2+^ displays the same effects as inhibition of mitochondrial Ca^2+^ release on PTP induction at MF synapses (Fig 4A–4D). Given the low affinity of the mitochondrial Ca^2+^ uptake system, mitochondria-associated ER membranes may thus play a critical role in the capacity of mitochondria to promptly respond to a change in cytosolic Ca^2+^ concentration and thereby affect many aspects of mitochondrial biology [55], including the control of exocytosis and/or endocytosis of synaptic vesicles.

Ca^2+^ ions play a versatile role in several steps of the cycle of synaptic vesicles through different Ca^2+^ sensors. Mitochondria thus affect exocytosis and endocytosis of synaptic vesicles through their ability to regulate Ca^2+^ homeostasis. However, the relative contribution of the ER and mitochondria to synaptic Ca^2+^-buffering appears to vary between different neuronal systems and might be influenced by the integration of different signaling pathways [56]. Previous studies in area CA1 suggested that intracellular Ca^2+^ release may have an effect on synaptic transmission. Depletion of ER Ca^2+^ stores or blockade of Ca^2+^-induced Ca^2+^ release reduces paired-pulse facilitation of excitatory postsynaptic potentials [46]. However, depletion of ER Ca^2+^ has no discernible effect at SC synapses on the magnitude of PTP (Fig 4E–4G), indicating that ER contribution to PTP induction is negligible at SC synapses. Indeed, thapsigargin, which depletes intracellular ER Ca^2+^ stores, has been shown to block long-term potentiation elicited by weak stimuli, but not that induced with strong stimuli, suggesting that calcium release from the ER plays a role in the regulation of synaptic plasticity [57].

## Conclusions

It has remained unclear whether hippocampal MF and SC synapses exhibit differential mechanisms of regulation of synaptic plasticity. In the current study, our quantitative EM analysis revealed large numbers of mitochondria at MF presynaptic terminals in contrast to very few mitochondria at SC presynaptic terminals. Reflecting the vast difference in mitochondrial content between MF and SC presynaptic terminals, the contribution of mitochondrial and ER Ca^2+^ to PTP induction and synaptic frequency facilitation is prominent at MF synapses but negligible at SC synapses. Taken together, these findings highlight the differences between MF and SC synapses in their regulatory mechanisms of short-term presynaptic plasticity.

## Supporting information

S1 Table Quantified data set of the number of mitochondria per cross-sectional bouton profile, the size of cross-sectional bouton profile area and the density of mitochondria. (DOCX)
