# Supplementary material for: Differential modulation of short-term plasticity at hippocampal mossy fiber and Schaffer collateral synapses by mitochondrial Ca2+
Source: PLoS One. 2020 Oct 13;15(10):e0240610. doi: 10.1371/journal.pone.0240610 (PMC7553293; doi:10.1371/journal.pone.0240610)
Supplement: S1 Table — (DOCX) [file pone.0240610.s001.docx]

| **Number of mitochondria per**  **cross-sectional bouton profile** | | **Cross-sectional bouton profile area**  **(µm^2^)** | | **Number of mitochondria per**  **µm^2^ of cross-sectional bouton area (mito/µm^2^)** | |
| --- | --- | --- | --- | --- | --- |
| **CA3** | **CA1** | **CA3** | **CA1** | **CA3** | **CA1** |
| 6 | 1 | 2.522 | 1.06 | 2.379064 | 0.9433962 |
| 8 | 0 | 3.835 | 0.211 | 2.08605 | 0 |
| 10 | 1 | 2.081 | 0.933 | 4.805382 | 1.071811 |
| 8 | 0 | 2.813 | 0.162 | 2.843939 | 0 |
| 10 | 0 | 2.608 | 0.191 | 3.834356 | 0 |
| 10 | 0 | 10.608 | 0.207 | 2.457002 | 0 |
| 8 | 1 | 3.256 | 0.199 | 1.264889 | 0 |
| 12 | 0 | 9.487 | 1.053 | 3.322259 | 0 |
| 15 | 0 | 4.515 | 0.28 | 2.378525 | 0 |
| 7 | 1 | 2.943 | 0.669 | 2.669953 | 1.494768 |
| 13 | 1 | 4.869 | 0.456 | 1.875533 | 2.192982 |
| 6 | 1 | 9.717 | 0.937 | 1.892915 | 1.067236 |
| 11 | 1 | 5.865 | 0.963 | 2.386635 | 1.038422 |
| 7 | 0 | 3.698 | 1.862 | 1.830384 | 0 |
| 8 | 1 | 3.352 | 0.772 | 1.882451 | 1.295337 |
| 9 | 1 | 4.917 | 0.661 | 1.103144 | 1.512859 |
| 9 | 1 | 4.781 | 0.853 | 2.565042 | 1.172333 |
| 10 | 1 | 9.065 | 1.531 | 1.287001 | 0.6531678 |
| 7 | 1 | 2.729 | 0.936 | 1.561199 | 1.068376 |
| 9 | 2 | 6.993 | 1.515 | 0.9056148 | 1.320132 |
| 15 | 0 | 9.608 | 0.795 | 6.269592 | 0 |
| 9 | 1 | 9.938 | 0.613 | 1.639728 | 1.631321 |
| 14 | 1 | 2.233 | 0.492 | 1.251324 | 2.03252 |
| 14 | 1 | 8.538 | 0.608 | 1.565435 | 1.644737 |
| 13 | 1 | 10.389 | 0.391 | 1.022059 | 0 |
| 15 | 1 | 9.582 | 0.309 | 3.654971 | 1.623377 |
| 12 | 1 | 11.741 | 0.616 | 1.23178 | 1.283697 |
| 5 | 1 | 1.368 | 0.779 | 3.757986 | 1.984127 |
| 12 | 1 | 9.742 | 0.504 | 1.00776 | 1.52207 |
| 10 | 1 | 2.661 | 0.657 | 4.370629 | 1.470588 |
| 10 | 1 | 9.923 | 0.68 | 1.484309 | 0 |
| 10 | 1 | 2.288 | 0.312 | 2.176542 | 2.832861 |
| 14 | 1 | 9.432 | 0.353 | 2.406015 | 2.247191 |
| 9 | 1 | 4.135 | 0.445 | 1.134282 | 0.5711022 |
| 8 | 1 | 11.075 | 1.751 | 1.592103 | 2.118644 |
| 8 | 1 | 3.325 | 0.472 | 2.467782 | 1.02459 |
| 13 | 1 | 11.461 | 0.976 | 4.716981 | 0.7256894 |
| 10 | 1 | 6.281 | 1.378 | 1.359722 | 0.5042864 |
| 9 | 1 | 3.647 | 1.983 | 2.603037 | 0 |
| 10 | 3 | 2.12 | 0.446 | 7.326007 | 0 |
| 9 | 0 | 6.619 | 0.639 | 6.267806 | 0 |
| 12 | 0 | 4.61 | 1.691 | 1.349406 | 1.396648 |
| 10 | 1 | 1.365 | 0.716 |  | 1.321004 |
| 11 | 1 | 1.755 | 0.757 |  | 1.048218 |
| 15 | 1 | 11.116 | 0.954 |  |  |
|  |  | 3.884 | 0.485 |  |  |
|  |  | 10.916 | 1.204 |  |  |
|  |  | 3.374 | 0.49 |  |  |

**Table S1. Quantified data set of the number of mitochondria per cross-sectional bouton profile, the size of cross-sectional bouton profile area and the density of mitochondria.**
